# The Contribution of Dance Movement Therapy in Promoting Nursing Students’ Interpersonal Skills during the COVID-19 Pandemic: A Descriptive Phenomenological Study

**DOI:** 10.3390/ijerph20021376

**Published:** 2023-01-12

**Authors:** Valerio Dimonte, Silvia Gonella, Beatrice Albanesi, Eugenia Malinverni, Sara Campagna, Alessio Conti

**Affiliations:** 1 Department of Public Health and Pediatrics, University of Torino, 10126 Turin, Italy; 2 Città Della Salute e Della Scienza University Hospital, 10126 Turin, Italy

**Keywords:** dance movement therapy, art-based education, COVID-19, relational skills, nursing students, professional education

## Abstract

During the COVID-19 pandemic, most universities closed or reduced clinical placements (CPs), limiting nursing students’ opportunities to practice communication and interpersonal skills before graduating. When applied in nursing curriculums, Dance Movement Therapy (DMT) enhances students’ understanding of the theoretical concepts of communication and interpersonal skills, representing a valuable educational tool when CPs are reduced, as during the COVID-19 pandemic. This descriptive phenomenological study aims to describe the contribution of DMT in promoting third-year nursing students’ relational skills during the COVID-19 pandemic. Thirty-four nursing students who attended a DMT workshop completed a reflective journal. Data were analysed using content analysis. Three themes emerged: struggling to care for patients during the COVID-19 pandemic, lived experience of DMT, and professional identity development. The first theme illustrates the connection participants made between their experiences during the DMT workshop and the caregiving challenges imposed by the pandemic; the second theme describes how the workshop fostered emotional and physical connections among its participants; the third theme focuses on the awareness participants acquired regarding their professional role during the workshop. When CPs opportunities are limited, DMT workshops can represent an educational tool to promote interpersonal and communication skills among nursing students, facilitating their transition into the profession.

## 1. Introduction

Successful transition from student to professional practice is a priority in educational programmes of healthcare professionals (HCPs) [1]. Without proper preparation and transition, the difficulties of adjusting to a real-life professional role can result in personal and professional vulnerability, especially in nursing students. Indeed, as nursing students move toward graduation, they may lack self-confidence and struggle to define their professional role [2]. Moreover, nurses transitioning into the profession may have poor communication skills [3] and be ill-equipped to deal with unexpected circumstances [4]. In HCPs’ education, the cognitive and technical domains of learning are often considered more relevant than the interpersonal domain, which, while contributing to the development of professional identity, is deemed to be related to the subjectivity of students [5].

For these reasons, experiential learning is a fundamental part of undergraduate HCPs curriculums, including hands-on and laboratory classes where students can develop their technical skills, and clinical placements (CPs) where they can put their acquired knowledge into practice [6]. The importance of experiential learning in promoting nursing students’ transition into the profession has been widely acknowledged and is reflected in the required hours of CP. Most healthcare educational institutions during the COVID-19 pandemic closed suddenly during the first wave in March 2020 and shifted their courses online to maintain social distancing and/or confinement policies [7]. Hundreds of thousands of students were forced to interrupt hands-on healthcare activities [8].

COVID-19 safety measures limited experiential learning opportunities, requiring schools to offer practical experience through alternative, safe, high-quality initiatives [9]. For example, the implementation of digital training, which achieved positive results, presented technical and organisational limitations and did not offer the real-time feedback that students receive during CPs [10]. Indeed, the hands-on practice and human interactions experienced during CPs are crucial in preparing nursing students for their transition into the profession and are challenging to deliver remotely [11]. In a report from Spain, undergraduate nurses in their final year of study who volunteered during the COVID-19 pandemic said that the experience allowed them to realise the importance of actively communicating with patients, and of interacting with the physical and social environment in which they will practice [12]. Hence, it follows that upon completion of their curriculum, it would be valuable to stimulate nursing students’ relational skills to prepare them for their professional role.

During the COVID-19 pandemic, universities focused on intervention-based teaching strategies and specific training programmes to prepare HCPs students for the health emergency, minimise their challenges, and facilitate their transition into the profession [13]. Simulation workshops have also been recommended to prepare future nurses and help them cope with their emotions during the pandemic [14]. However, few studies describe interventions meant to enhance interpersonal skills among nursing students finishing their undergraduate education, particularly in a context where hands-on training and CPs are limited. Learning experiences focused on developing interpersonal skills could represent a resource for nursing education in promoting professional identity acquisition that could be complementary to CPs and different hands-on laboratory classes, increasing the educational opportunities available to HCPs students.

Dance Movement Therapy (DMT) is an art-based strategy that can assist in developing interpersonal skills and has been shown to allow first-year undergraduate nursing students to apply relational and communication skills and strengthen their understanding of theoretical concepts [15]. DMT uses a series of specific interventions that promote biopsychosocial development by incorporating it into the recreational and aesthetic properties of dance [16]. DMT has been shown to have several benefits to mental health (e.g., reducing depression and anxiety) and wellbeing (e.g., enhancing interpersonal skills and quality of life) [17]. That is because DMT, particularly relational expressive DMT (DMT-ER^®^), emphasises the individual’s integration into a group using a ludic approach, making it especially suitable for educational purposes [18].

DMT represents a tool that can enable nursing students to develop and hone their interpersonal skills, including the core nursing competencies of communication and relational skills [19]. This could offer an opportunity to foster such skills in nursing students at the end of their undergraduate curriculum, which, combined with CPs, can facilitate students’ transition into the profession and a recognition of their professional identity. This study aimed to describe the contribution of DMT in promoting third-year nursing students’ relational skills during the COVID-19 pandemic.

## 2. Materials and Methods

### 2.1. Study Design

This study followed a descriptive phenomenological approach [20,21], which implies a direct analysis and exploration of a particular phenomenon. In descriptive phenomenological methods, researchers are not required to make interpretations, but rather analyse participants’ descriptions to gather the essential meaning characterising their experiences. This contributes to clarity and the understanding of the phenomenon of interest from the perspective of those directly engaged in it [22]. Thus, this method was chosen to produce a comprehensive description of the enhancement of nursing students’ communication and relational skills in relation to their lived experience during DMT workshops.

### 2.2. Context

Italy was the first European country to report a COVID-19 outbreak. The northwest regions were the most severely impacted by the first wave of the pandemic [23], during which several universities suspended classes and CPs to limit the spread of infection. At the end of the first wave (May 2020), third-year undergraduate nursing students at the University of Torino could attend voluntary CP in limited settings (e.g., pre-hospital triage, contact tracing services, and psychiatric outpatient services). In addition to issues related to skill acquisition and retention, these limitations meant that many nursing students did not reach the number of CP hours required to graduate, and caused a large number of HCPs to enter the profession later than they should have [24].

### 2.3. The Dance Movement Therapy Workshop

The DMT workshop in this study was explicitly developed, in collaboration with the Italian School of DMT-ER, as an educational opportunity targeted at fostering interpersonal skills in third-year undergraduate nursing students who, due to the COVID-19 pandemic, were not able to complete the number of CP hours required to graduate. The University of Torino has employed DMT methodology with first-year undergraduate nursing students since 2015/2016 to develop communication and relational skills [15].

**Figure 1:**
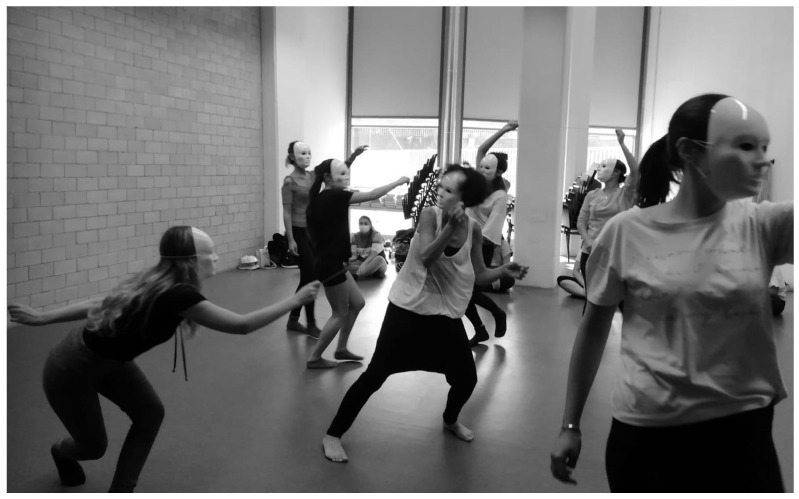
Participants to the DMT workshop wearing neutral masks.

The workshop took place in October 2020 and consisted of one 6 h session. Two certified dance therapists, who were also nurses, conducted the workshop, which included games with escalating complexity to facilitate comprehension of the concepts of distance and closeness in communication, and to promote reflection on the nursing profession during the COVID-19 pandemic. Each student was provided a plastic, neutral, white mask, which hid facial expressions (Figure 1), and a piece of cotton string measuring 1 m. Students were sometimes divided into groups, with one group actively playing the game and the other observing.

The games and their respective learning objectives are presented in Table 1. Safety protocols were respected at all times: participants maintained silence during the games and kept at least 1 m of distance between themselves and used personal protective equipment. Neutral masks were disinfected after each 6 h session.

Students received an e-mail 2 weeks before the DMT workshop, with detailed information about the workshop and the purpose for including it at the end of their curriculum. Attendance to the DMT workshop was required, but participation in the games was voluntary; students who wished could simply observe, and those who chose to participate were allowed to leave the games at any time. Conductors observed students’ reactions to detect any emotional issues, and the workshop began and ended with low-emotional-impact games, while the most intense activities occurred in between. Students were divided into groups of 15–17. Each game lasted 1–1.5 min, with verbal debriefing and ritual dances used as safe ways to elaborate on and exit from the games.

### 2.4. Setting and Participants

Data were collected at two locations of the University of Torino during the 2020/2021 academic year. A total of 48 third-year undergraduate nursing students, all of whom had yet to reach the number of CP hours required to graduate, attended the DMT workshop. All attendees had already participated in DMT workshops during their first year of study. Participants were mostly females (n = 38, 79%; median age: 23 years, range 21–25).

### 2.5. Data Collection

At the end of the DMT workshop, participants were asked to reflect on and record their experience in a reflective journal, intended as a written narrative record of their learning experience [25]. Following a series of open questions, developed by the researchers as a topic guide, they were asked to describe: (1) their physical feelings and emotions during the workshop, (2) their perspectives in the observer role, and (3) the connections between their experience during the workshop and the professional nursing role. No word limit was set; participants were informed that reflective journals were optional, that they could deny consent to use their journals, and that the journal would only be used for research purposes and did not constitute part of their grade. They were allowed to use informal language to describe their learning experience. Reflective journals elicit critical thinking and allow participants to increase their awareness and broaden the quality of their learning [25].

At the University of Torino, nursing students often use reflective journals, particularly after CPs or workshops. In particular, participants were previously trained in reflective journal writing when they attended their first year DMT workshop [15]. This data collection method was preferred to limit the risk of COVID-19 infection while ensuring the privacy of the students and the free expression of their thoughts. Participants were asked to complete and return the reflective journals via e-mail within 3 days of the DMT workshop; 34 (71%) returned a journal and gave permission for its use. Journals were stored on a secure university cloud service. The median length of reflective journals was 881 words (range 675–1322).

### 2.6. Data Analysis and Rigour

Data were analysed according to the descriptive phenomenological approach [20,21]. Researchers immersed themselves in the phenomena of interest by reading participants’ experiences several times. This allowed them to obtain a sense of the whole, avoiding using their previous personal evaluations and opinions for interpretive purposes [26]. Bracketing their foreknowledge is a fundamental assumption of the descriptive phenomenological method [27], which researchers exercised through deep reflection on their prior experiences and preconceptions, potentially affecting their understanding of data. Subsequently, an inductive content analysis [28] was performed independently by two researchers (AC, SG) who read the reflective journals carefully, coded the participants’ sentences that were condensed into meaning units according to their content, and then grouped these meaning units into categories. These categories were grouped into themes, and the themes were labelled in accordance with their content, and then listed and analysed to identify any connections between them. Researchers reached a consensus on the themes; in case of disagreement, a senior researcher (VD) was consulted. Two independent auditors (BA, SC) reviewed the final categories and themes. Rigour was ensured through an audit trail, peer debriefing, and reflexivity through journaling during data analysis and interpretation [29]. Reflective journals were written in Italian; quotes were translated into English for publication purposes.

### 2.7. Ethical Consideration

The ethical review and approval were waived for this study. The COVID-19 pandemic resulted in an interruption of CPs and an urgent need for universities to offer alternative educational strategies, ensure that nursing students obtain adequate interpersonal skills and experience, and allow them to complete the CP hours required for their graduation. To respond promptly to this educational need and fill this educational gap, the University of Torino proposed the Dance Movement Therapy workshop. Among the activities that comprised the workshop, writing a reflective journal was a fundamental step in completing the workshop. Consequently, we cannot produce more information on the Ethics Committee’s name, approval code, and date. However, we ensured that all applicable institutional and governmental requirements concerning the ethical use of human volunteers were followed. Data were collected with respect to the principles of the Helsinki Declaration. The anonymity and confidential treatment of the data was maintained during analysis and reporting. Students were informed about the aim of the study and its procedures before they completed the reflective journal. Each reflective journal was given a sequential identification number, which was then anonymised. Anonymity and confidential treatment of data were maintained during analysis and reporting. 

## 3. Results

**Figure 2:**
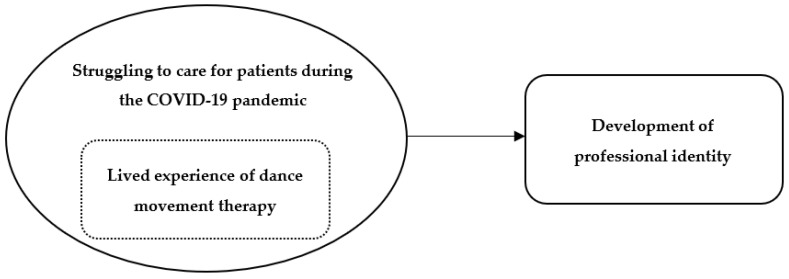
Visual representation of themes.

Three themes emerged: 1. struggling to care for patients during the COVID-19 pandemic, 2. lived experience of DMT, and 3. development of professional identity. The first theme illustrates the connection participants made between their experiences during the DMT workshop and the caregiving challenges imposed by the COVID-19 pandemic; the second theme describes the characteristics of the workshop that fostered emotional and physical connections among its participants; and the third theme focuses on participants’ acknowledgement of the awareness they acquired regarding their future professional role during the workshop (Figure 2). Each reflective journal (i.e., each participant) contributed to all themes (Supplementary Material, Table S1). The first theme was the most grounded, with 8 categories and 563 primary codes. It was followed by the third theme, with 7 categories and 313 primary codes, and then the second, which contributed 5 categories and 193 primary codes. For each reflective journal, a median of 31 [minimum–maximum 17–47] primary codes were identified. Nine reflective journals had a number of codes equal to or greater than the third quartile.

### 3.1. Struggling to Care for Patients during the COVID-19 Pandemic

Participants described how the DMT workshop caused them to reflect on caregiving difficulties during the COVID-19 pandemic. Specifically, students emphasised that the surgical and neutral masks represented barriers to communication, which epitomised the challenges experienced by HCPs whose entire bodies were covered with personal protective equipment. Although the use of masks and silence during the workshop presented limitations with regard to facial expressions and verbal communication, participants highlighted that these devices allowed them to focus on their peers’ movements as a way to understand their feelings.

*“With barriers such as masks or protective gowns that HCPs had to wear during the emergency, verbal communication was severely limited. However, this situation allowed nurses to develop observational skills, just as in our [DMT] setting it allowed us to observe our colleagues dancing”*. (9:10)

The precautions taken during the DMT workshop precluded physical contact, so participants inevitably reflected on the concepts of proximity and distance. Participants emphasised how difficult it was not to be able to get close to their peers, likening it to the feelings that nurses experienced during the COVID-19 pandemic. Nevertheless, students acknowledged that the games helped them realise that they could get physically closer if they maintained the prescribed distance, and still establish an emotional closeness. They compared this process to the nurse–patient relationship, and how the distance one needs to maintain does not entirely hinder contact.

*“In this sense, I perceived it [the distance] as a limitation; because even if you tried to get closer, it was impossible to do it completely. […] I find that to be relevant to this period, as keeping distance between people has also changed the way we relate with others. In healthcare, it makes me think that HCPs being all covered up somehow had a ‘wire’ that limited their proximity to patients”*. (10:4)

The DMT workshop helped participants recall experiences from their CPs during the COVID-19 pandemic; they described a sense of depersonalisation, communication difficulties, and difficulties transmitting closeness, comparing them to the feelings they perceived during the games. Participants realised that they used the same tools to facilitate communication in their CPs as during the workshop and identified the games as a means to reframe their experience in a safe environment.

*“This workshop reminded me of my last placement […] at the psychiatric outpatient clinic; […] I really found it difficult to interact with patients because of the mask, which made it difficult, especially for the patient, to interpret emotions. Many of these patients already struggled to express themselves, so the masks created another barrier […], which we tried to overcome through eye contact and the tone of our voice to make the other person feel close”.* (18:8)

### 3.2. Lived Experience of Dance Movement Therapy

It was natural for participants to compare the third year DMT workshop with their previous experience as first-year students. While students found the games from both workshops to be similar, they still reported a sort of emotional bewilderment, primarily due to the total absence of physical contact and the silence imposed throughout the experience.

*“On Wednesday, I attended the DMT workshop, and I have to say that it was a similar session to those I had previously attended, but emotionally it was quite different”.* (33:1)

Participants’ narratives emphasised that the familiarity and solidarity they had developed with their peers helped them frame their emotions during the DMT workshop. The increased knowledge and intimacy students had with each other allowed them to limit considerably the sense of discomfort they sometimes perceived during the first-year workshop. Specifically, their confidence in their peers, combined with the suspension of judgment, allowed participants to develop relationships that affected them physically and emotionally.

*“Two years have passed since the first meetings [DMT workshop], so it was inevitable for me to compare the two experiences; especially the emotional perspectives, which were […] different. Perhaps what differentiated them was the greater knowledge and complicity that we [the participants] had. […], I did not feel the same discomfort as in the previous meetings”.* (22:1)

### 3.3. Development of Professional Identity

The DMT workshop allowed participants to reflect critically on the historical moment, and on the changes that the COVID-19 pandemic imposed on their therapeutic relationships with patients. Through the games, they were able to identify the human and professional evolution they had undergone over their 3 years of study. Some participants noted an improvement in their communication skills.

*“I think that the workshops, the one in the first year before the first internship, and then repeated in the third year at the end of the undergraduate course, make you aware of the progress you have made on a human level. […] it makes you understand how you have changed, and how your way of communicating with patients has changed, [...], it [communication] is fundamental in the nurse-patient relationship”.* (8:12)

Participants described how the DMT workshop promoted their awareness of their own and their peers’ bodies, including a consciousness of others’ time and space and how to respect these. In this sense, students identified the dance as an effective metaphor to describe nurses’ caregiving relationship with patients.

*“All of this, applied in nursing practice, is meant to convey the message of the caring relationship, that bond that is created between the nurse and the patient, that dance that varies continuously: on tiptoe, slowly, quickly, firmly, and gently. In such a relationship, you have to be aware of when you should stand still and observe, wait and recognise the kind of person you have in front of you—their way of thinking, their culture, their beliefs—wait for the other person to take the first step, have the ability to say and do things at the right time, and accompany them in the phases of their life, fit into their dance and share it”.* (32:7)

Participants also discussed the value of observing their peers playing and likened that outside experience to those they lived during their CPs. They expressed how their empathic skills were strengthened when they put themselves in their patient’s shoes. Participants saw their peers take responsibility for each other, thereby acknowledging the commitment to understand and respect patients’ needs.

*“As an ‘observer’, I could understand the feeling of helplessness that patients sometimes have. Individuals do not always feel that they are part of their care pathway; they see HCPs act, but they don’t have time to stay on top of every single aspect that concerns them, and they feel left behind by every new event”.* (20:5)

Participants described how, during the games, their attention progressively focused on their own and their peers’ body language. The DMT workshop helped them understand the value of non-verbal communication and its importance in the nursing profession. Nursing students understood how necessary comprehensive communication skills are if they want to successfully connect and be close with patients who might feel distanced from them.

*“I also realised that we need to pay more attention to our body language, because even the tension or relaxation of a muscle can help us understand if a person is comfortable or not […]. A large part of communication nowadays is non-verbal, but through a person’s body and eyes you can understand their intentions and what they want to communicate”.* (18:5).

## 4. Discussion

The aim of this study was to describe the contribution of DMT in promoting third-year nursing students’ relational skills during the COVID-19 pandemic. Our findings emphasised how, in a scenario characterised by education and communication challenges due to external contextual factors, the DMT workshop helped nursing students enhance their interpersonal skills, promoting their professional identity development.

The DMT workshop allowed participants to reflect on their undergraduate CPs. The COVID-19 pandemic provoked profound changes in nursing care [23], which nursing students witnessed in their fragmented CPs. They experienced a reduced possibility to communicate with patients and colleagues due to personal protective equipment and social distancing, which contributed to anxiety over infection [12]. The DMT workshop reminded participants of their real-world experiences during the first wave of the COVID-19 pandemic, which was characterised by communication challenges. The workshop constituted a resource that enhanced students’ communication and interpersonal skills beyond contextual challenges, thus facilitating their definition of nursing professional identity. In this connection, the neutral mask used during the games was primarily intended to emphasise body centrality and embodied learning, but also allowed participants to reflect on nursing identity.

Corporeality plays a leading role in DMT workshops, allowing for embodied learning—both cognitive and experiential—through dance [17,18]. The central role of the body is fundamental in medical training, as it enables the development of reflection and self-awareness [30], which are difficult to practice during public health emergencies. Furthermore, empathic and reflective skills are essential to HCPs [31], and nursing students’ accelerated transition into the profession during the COVID-19 pandemic may have negatively impacted these skills [12]. In this sense, the DMT workshop exposed participants to a real-life situation, allowing students to experience interpersonal skills even in a situation of limited CPs.

Participants appreciated their educational experience and recognised their maturation compared to their first year DMT workshop. In this perspective, DMT relies on principles of graduality and interaction among group members [18] that could be useful in nursing curricula to promote confidence in one’s peers and enhance group identity, especially during healthcare emergencies. The COVID-19 pandemic has led to an acknowledgement of the benefits of interprofessional teamwork, which is a valuable skill for all nurses [12]. The first-year DMT workshop promoted communication skills and enhanced participants’ understanding of the theoretical concepts of communication and relational skills [15], but for third-year students, it contributed also to the transition into the nursing profession. This suggests that repeated DMT may promote teamwork skills among nursing students, and students of other healthcare professions, and thus may be a means to foster interprofessionalism.

This educational experience conducted during the COVID-19 pandemic could demonstrate how anticipating and re-experiencing real-life situations in a protected environment can alleviate psychological symptoms in students, shielding them from the clinical reality while allowing the practical application of theories delivered in the classroom [11]. In this connection, the nursing curriculum teaches interpersonal skills, such as ethical commitment which has been fundamental during the COVID-19 pandemic, through their mobilisation in practice [12]. DMT has already been recognised as a tool that can be applied in nursing education to anticipate theoretical concepts related to interpersonal skills, such as modulation, active listening, and body language [15], representing a resource when CPs are limited.

Reflection, commitment, and accountability have already been identified as crucial components of professional identity development [32,33], and participants reported these as characterising aspects of the DMT workshop. Indeed, they confirmed the value of the first-year DMT workshop in promoting reflection on professionalism [15], and reported gaining awareness of their human and professional development over their 3-year undergraduate curriculum. As those studying to become HCPs commonly show limited preparedness for their professional role [34], DMT could be a valuable tool in helping participants reflect on and reframe professional roles, promote self-awareness and, in turn, professionalism.

### Strengths and Limitations

This is a single-centre study involving a limited number of participants. Reflective journals were preferred for safety and logistical reasons. The journals allowed for broader data collection, but not the depth of understanding provided by in-depth interviews or focus groups. Moreover, it could be possible that participants who decided to write their reflective journal had different feelings, experiences, or were more motivated than those who declined participation. Nevertheless, a rigorous methodology, data collection, and analysis consistent with the aim of the study were applied. Rigour was ensured through the use of an audit trail, journaling and reflexivity, and peer debriefing. The dance therapists who conducted the workshop were not part of the research team, which limited the possibility of social desirability bias. Moreover, students were constantly assured that their journals were only used for research purposes. The generalisability and assessment of the effectiveness of DMT can be demonstrated only through quantitative studies applying longitudinal or experimental designs.

## 5. Conclusions

This study provides qualitative evidence on the possibility for universities to consider introducing DMT methodology when CP opportunities are limited, as it may represent a viable educational instrument fostering interpersonal and communication skills in nursing students. A considerable body of concepts that characterise professional nursing identity was explored and reinforced through the DMT workshop, enabling the creation of an easily identifiable link between theory and practice. Adopting a reflective approach allowed participants to acknowledge the progress they had made during their undergraduate curriculum on both a personal and professional level, facilitating their professional development. Future studies adopting quantitative design could corroborate the obtained results, investigating the contribution of this educational method to the development of group identity in nursing students, and the positive effect on appraising students’ interpersonal skills and self-awareness if repeated DMT workshops are offered as part of the undergraduate curricula of HCPs.

## Figures and Tables

**Table 1:** Organisation of the Relational Expressive Dance Movement Therapy (DMT-ER) third-year workshop, description of the games and their objectives.

Name	Objectives	Description
**Presentation of the workshop**	Setting the stage and explaining the rules of the workshop.	Everybody sits in a circle on the floor. The conductors explain the rules of the workshop and the safety devices.
**Game of the names** **You, you, me, me**	Familiarisation with the group and memorisation of names. Creating the group by learning names.	Everyone, in succession, recites the name of the person on their right, then does the same for the person on their left. The rest of the group repeats this last name in unison.
**Segmented semi-structured warm-up, Stop and Go**	Develop movement working with patterns, such as core-distal, spinal, homologous, homolateral, cross-lateral, and effort quality.	Participants walk around the room slowly, then more quickly, Stop and Go; move and play exploring movements with upper or lower body, trunk, and so on, till they have played with all parts of the body, sometimes with another participant (maintaining social distancing) they meet in the room.
**Introducing the neutral mask**	Familiarisation with the neutral mask.	Participants walk around the room and stop while wearing the neutral mask; then they remove the mask and start walking around again. After a first familiarisation with the device, they wear the neutral mask and are allowed to walk around the room and move their bodies.
**The string of relationship**	Exploring relationships, diversity of styles, bodies, the constraint represents the possibility to communicate.	Participants play with the colours of the strings, forming pairs. Participants wear the neutral mask and dance, holding the string with two fingers and keeping it taught. Pairs change throughout the game. Half of the group is in the observer role. The game ends with a dance of both subgroups, without wearing neutral masks.
**Debriefing**	Debriefing.	In a circle, everybody shares their reflection on the experience.
**Dance of the four directions**	Giving a sense of closure to the experience.	Everybody performs the ritual dance of the four directions.
**Pause**		
**Semi-structured warm-up**	Re-start and prepare bodies for other games.	Participants move their bodies and play with other participants, discovering closeness and distance (maintaining social distancing), developing human networks. Then, participants stop, close their eyes for a few seconds; repeat that a few times, make eye contact with one person, and form pairs.
**Rhythmic imitative warm-up**	Mirroring. Gradual transformation of the movements. Identification/differentiation. Experience leadership and unity.	Everybody wears the neutral mask and forms a line. The first person in line makes a gesture, and everyone else must repeat it. At the beginning, the gestures are proposed by the conductor, then participants themselves take turns leading the line, and each proposes their own gesture. The game ends with everybody proposing a gesture without wearing the neutral mask.
**The plaster cast collection**	Mirroring. Understanding the reciprocity of movements. Identification/differentiation.	Participants wear the neutral mask and are divided into two groups: one playing the game (performing group) and the other in the observer role. Half of the performing group lock their bodies in a certain position like a plaster cast. The other half of the performing group moves around freely. They then choose a locked peer and to mimic, which unlocks the locked peer, but locks themselves into a position that is slowly changed, until they become a new plaster cast. Participants can shift their role as they want throughout the game. The game ends with a plaster cast collection formed by both subgroups, a first time wearing the neutral mask, finally without wearing it.
**Debriefing, one word to say it**	Debriefing.	In a circle, everyone shares their reflection. Everybody chooses one word to describe their experience, writes it on a white piece on paper, and explains it to the whole group.
**Dance of the four directions**	Giving a sense of closure to the experience.	Everybody performs the ritual dance of the four directions.

## Data Availability

The data presented in this study are available on request from the corresponding author. The data are not publicly available due to privacy restrictions.

## Supplementary Materials

The following supporting information can be downloaded at: https://www.mdpi.com/article/10.3390/ijerph20021376/s1, Table S1: Frequencies of codes provided by participants to each category of the three themes.
