# Supplementary material for: The Contribution of Dance Movement Therapy in Promoting Nursing Students’ Interpersonal Skills during the COVID-19 Pandemic: A Descriptive Phenomenological Study
Source: Int J Environ Res Public Health. 2023 Jan 12;20(2):1376. doi: 10.3390/ijerph20021376 (PMC9858882; doi:10.3390/ijerph20021376)
Supplement: Supplementary file 1 [file ijerph-20-01376-s001.zip › ijerph-2040914-supplementary.pdf]

Table S1. Frequencies of codes provided by participants to each category of the three themes

Participant	Theme 1. Struggling to care for patients during the COVID-19								Theme 2. Lived experience of Dance Movement Therapy					Theme 3. Development of professional identity							
	Categories								Categories					Categories							
	Connection with the reality	Neutral mask	Surgical mask	Eye contact	Facial expression	Proximity / Distance	Adaptation	Reframing experience safely	Comparison with the first DMT workshop	Group	Harmony / Balance	Silence	Touch	Body perception	Body awareness	Reflecting on the nursing role	Modulation / Proxemics	Personal development / DMT contribution	Clinical Placement	Utility	**Total codes**
**#1**	2	2	0	0	0	5	4	3	1	1	1	1	1	1	1	1	1	2	1	1	**29**
**#2**	3	1	1	1	1	1	2	2	0	0	0	0	1	2	0	0	1	0	0	3	**19**
**#3**	0	1	1	1	0	3	6	1	0	0	1	0	0	4	1	0	0	1	0	0	**20**
**#4**	2	3	2	1	1	2	2	4	1	0	3	0	0	5	1	1	3	2	1	2	**36**
**#5**	1	0	2	0	3	3	4	3	0	0	3	1	0	4	2	0	2	1	1	1	**31**
**#6**	0	2	0	0	2	6	6	5	1	1	5	1	0	5	2	2	2	2	1	0	**43**
**#7**	2	3	1	1	2	4	1	6	2	2	4	1	0	3	3	0	2	1	0	0	**38**
**#8**	4	2	1	2	1	2	3	1	3	1	5	0	3	2	3	0	1	2	1	4	**41**
**#9**	2	2	2	1	2	0	4	6	1	2	7	1	1	1	1	0	1	0	0	1	**35**
**#10**	4	3	2	0	3	8	2	5	0	1	7	2	0	6	0	2	0	1	1	0	**47**
**#11**	0	4	2	1	1	3	1	7	0	1	5	1	1	2	3	0	2	2	1	1	**38**
**#12**	0	1	1	2	0	3	1	8	0	2	7	0	0	5	3	1	1	2	0	1	**38**
**#13**	3	3	1	1	2	7	3	5	0	1	6	2	1	3	1	1	2	2	0	0	**44**
**#14**	1	3	0	1	0	4	1	5	1	0	4	0	1	2	2	2	3	0	0	1	**31**
**#15**	4	2	2	0	1	5	1	4	3	1	6	0	1	4	1	2	2	2	2	2	**45**
**#16**	0	3	2	2	0	6	1	6	1	3	4	1	1	6	2	1	0	2	0	2	**43**
**#17**	5	3	5	2	2	2	2	3	2	1	3	1	0	3	0	1	0	0	1	1	**37**
**#18**	4	1	3	2	0	2	3	1	0	0	2	1	0	1	0	2	0	2	1	4	**29**
**#19**	1	1	3	0	1	3	2	3	1	1	4	0	0	2	1	1	0	1	0	0	**25**
**#20**	1	2	1	1	1	3	1	4	1	1	3	0	1	0	1	1	0	2	0	2	**26**
**#21**	2	4	2	1	2	4	1	5	0	0	3	0	1	2	3	0	1	1	0	1	**33**
**#22**	2	2	2	3	1	2	1	4	1	1	1	0	1	3	0	2	0	0	0	1	**27**
**#23**	3	1	1	3	1	2	1	2	0	0	2	0	0	2	0	2	1	3	2	1	**27**
**#24**	4	1	2	1	1	1	0	1	1	0	2	0	0	0	0	0	0	2	1	2	**19**
**#25**	4	2	0	0	4	0	1	2	1	0	0	1	0	3	0	4	0	3	0	4	**29**
**#26**	3	2	2	1	2	4	0	4	1	0	1	0	2	3	2	0	0	1	0	1	**29**
**#27**	4	2	2	1	0	5	1	6	1	1	3	0	1	1	0	3	0	2	1	3	**37**
**#28**	2	1	2	0	0	3	1	2	0	0	2	0	1	1	0	0	0	1	0	1	**17**
**#29**	4	1	2	1	2	1	1	1	0	0	2	0	0	0	0	2	0	4	0	5	**26**
**#30**	1	4	3	1	0	5	2	4	0	0	2	0	0	3	0	0	0	1	0	0	**26**
**#31**	1	1	0	1	0	1	0	1	1	0	1	0	0	1	0	1	1	1	0	1	**12**
**#32**	3	1	1	0	0	5	2	3	0	0	6	0	1	2	3	1	0	4	0	3	**35**
**#33**	2	2	2	1	1	0	0	1	1	0	2	0	0	3	0	0	0	3	0	3	**21**
**#34**	1	4	3	1	1	1	1	4	0	1	5	0	1	5	0	1	0	3	1	3	**36**
**Total codes for each category**	**75**	**70**	**56**	**34**	**38**	**106**	**62**	**122**	**25**	**22**	**112**	**14**	**20**	**90**	**36**	**34**	**26**	**56**	**16**	**55**	**1069**
**Total codes for each theme**	**563**								**193**					**313**							**1069**

Abbreviations: DMT= Dance Movement Therapy
